# Mixed Warm and Cold Autoimmune Hemolytic Anemia With Concomitant Immune Thrombocytopenia Following Recent SARS-CoV-2 Infection and Ongoing Rhinovirus Infection

**DOI:** 10.7759/cureus.38509

**Published:** 2023-05-03

**Authors:** Frederic Karim, Kalsi Amardeep, Aaron Yee, Benjamin Berson, Perry Cook

**Affiliations:** 1 Internal Medicine, NewYork-Presbyterian Brooklyn Methodist Hospital, Brooklyn, USA; 2 Hematology and Oncology, NewYork-Presbyterian Brooklyn Methodist Hospital, Brooklyn, USA; 3 Pulmonary and Critical Care Medicine, NewYork-Presbyterian Brooklyn Methodist Hospital, Brooklyn, USA

**Keywords:** immune thrombocytopenia (itp), viral itp, paxlovid, post covid-19 aiha, mixed aiha, autoimmune hemolytic anemia (aiha)

## Abstract

Mixed-type autoimmune hemolytic anemia (AIHA) is a term used to describe hemolysis occurring in the context of both warm and cold reactive autoantibodies to red blood cells. Immune thrombocytopenia (ITP) is an acquired form of thrombocytopenia potentially complicated by hemorrhage due to autoantibodies reactive with platelets and megakaryocytes. Diagnosis of ITP requires exclusion of other known causes of thrombocytopenia. AIHA and ITP may be primary disorders or associated with lymphoproliferative, autoimmune, or viral infections. Here, we report a rare case of simultaneous mixed-type autoimmune hemolytic anemia with immune thrombocytopenia following severe acute respiratory syndrome coronavirus 2 (SARS-CoV-2) infection treated with Paxlovid followed by Rhinovirus infection.

## Introduction

Coronavirus disease 2019 (COVID-19) has been associated with multiple hematologic conditions, such as immune thrombocytopenia (ITP) and autoimmune hemolytic anemia (AIHA). New-onset ITP associated with COVID-19 has been described in the literature. Many of these cases (50%) occurred in patients over 50 years old [1]. Additionally, exacerbation of chronic or persistent ITP has been reported following COVID-19 [2]. The onset of ITP typically occurred 2-3 weeks following COVID-19 infection. Most COVID-associated ITP responded to standard ITP therapy with intravenous immunoglobulin (IVIG), glucocorticoids, or both. AIHA can be categorized as cold, warm, or mixed AIHA. Common infections associated with cold AIHA include mycoplasma pneumonia, cytomegalovirus, mumps, varicella, adenovirus, HIV, influenza A, and hepatitis C virus. On the other hand, conditions associated with warm AIHA include systemic lupus erythematosus, immune deficiency syndromes, leukemia, lymphomas, and infections [3]. COVID-19 has been associated with cold, warm, and mixed AIHA. Onset occurs a median of nine days after COVID diagnosis with a range of 4-13 days [4]. The first-line treatment for warm AIHA is corticosteroids with or without rituximab. For persistent disease, many cytotoxic options have been described including cyclophosphamide, mycophenolate, azathioprine, danazol, cyclosporine A, sirolimus, and plasma exchange [5]. In contrast to warm AIHA, cold AIHA does not respond to steroids, and the first-line treatment should include rituximab or sutimlimab with subsequent cytotoxic drugs or Bruton tyrosine kinase inhibitors [6]. Some of the cytotoxic drugs used are bendamustine or fludarabine. Mixed AIHA is diagnosed by direct antiglobulin testing showing both a pattern of IgG and complement C3, as cold agglutinins.

## Case presentation

A 30-year-old male with a medical history of alcohol use disorder and confirmed COVID-19 infection by nasal polymerase chain reaction (PCR), treated with five days of Paxlovid, presented with two days of fevers, nasal congestion, jaundice, oral and lower extremity petechiae, fatigue, and dyspnea on exertion. He reported a recent trip to Greece and upstate New York in the last two months. His roommate had a reported minor upper respiratory infection. The patient reported no previous influenza vaccination but received three Moderna mRNA-1273 vaccine doses, and the last booster shot was given six months before admission. There was no contributing personal or family history of blood disorders or cancers and no history of unprotected sex.

On admission, the patient presented with hypertension, tachycardia, and fever. The viral respiratory panel was positive for Rhinovirus/Enterovirus and negative for severe acute respiratory syndrome coronavirus 2 (SARS-CoV-2). Hematologic testing revealed a white blood cell count (WBC) of 5.33 x 103 cells/µL and severe anemia with hemoglobin (Hgb) levels of 3.9 g/dL, a mean corpuscular volume (MCV) of 143 fL, and a platelet count of 1 x 103 cells/µL. Additional lab work demonstrated low haptoglobin levels (<7.8 mg/dL), high lactate dehydrogenase levels (783 units/dL), high fibrinogen activity (593 mg/dL), an international normalized ratio of 1.1, an activated partial thromboplastin time of 22.6 seconds, a prothrombin time of 12.6 seconds, and ADAMTS13 activity of 77%. Direct antiglobulin testing was positive for serum IgG and C3. Complement levels were also assessed, with C3 levels at 85 mg/dL and C4 levels at 11 mg/dL. Total bilirubin was elevated at 3.0 mg/dL, while direct bilirubin was within range at 0.4 mg/dL.

**Figure 1:**
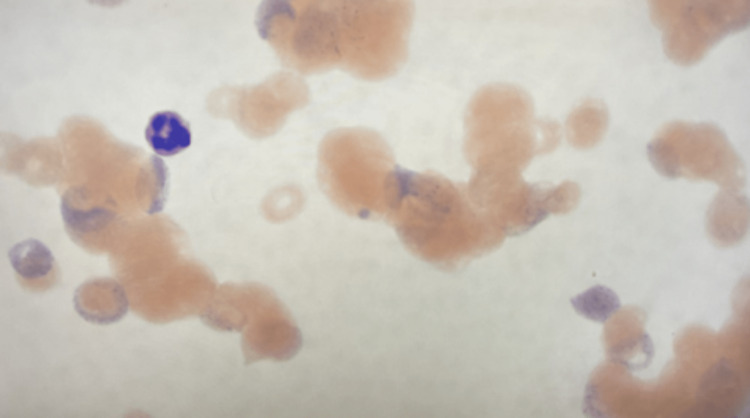
Peripheral smear (prior to warming) showing red blood cell clumping and agglutination.

**Figure 2:**
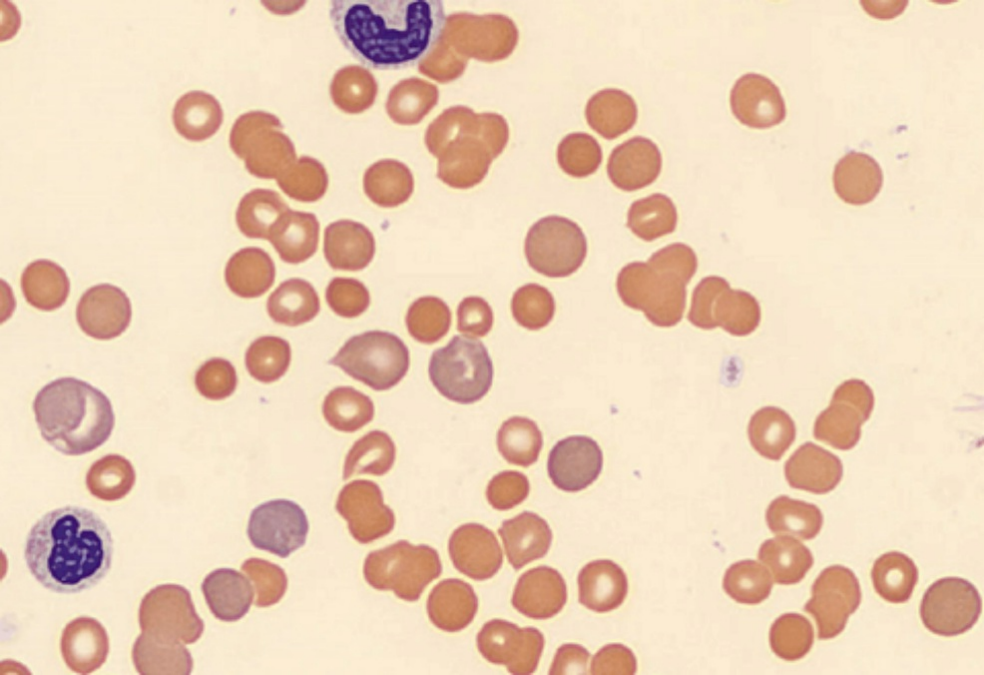
Peripheral smear showing de-agglutination following warming of the hematologic sample.

Peripheral smear demonstrated red blood cell (RBC) clumping with increased reticulocytes, few platelets, and no schistocytes (Figure 1). Subsequent warming of the hematologic sample revealed a Hgb of 12.6 g/dL with an MCV of 139 fL, a WBC of 2.06 x 103 cells/µL, and platelets of 2 x 103 cells/µL, along with de-agglutination (Figure 2). Testing for hypercoagulable state and autoantibodies was negative (Table 1). Hepatitis serology was positive for hepatitis B core antibody and hepatitis A total antibody; further viral serology workup was negative (Table 2). Additionally, the patient underwent a lymphoproliferative workup. Peripheral flow cytometry showed no evidence of clonal B cells, and CT chest, abdomen, and pelvis with IV contrast did not show any adenopathy, organomegaly, or pneumonia.

**Table 1:** Coagulation studies and hemolysis workup. INR, international normalized ratio, Ig, immunoglobulin.

Biochemical parameter	Patient values	Reference range
Haptoglobin	< 7.8 mg/dL	30.0 - 200.0 mg/dL
Lactate dehydrogenase	783 unit/dL	87 - 241 unit/L
Fibrinogen	593 mg/dL	276 - 471 mg/dL
INR	1.1	0.9 - 1.1
Activated partial thromboplastin clotting time	22.6 seconds	25.1 - 36.5 second (s)
Prothrombin time	12.6 seconds	9.4 - 12.5 second (s)
Creatinine	0.8 mg/dL	0.67 - 1.17 mg/dL
Urea nitrogen	13 mg/dL	7 - 18 mg/dL
Total bilirubin	3 mg/dL	0.3 - 1.0 mg/dL
Direct bilirubin	0.4 mg/dL	0 - 0.3 mg/dL
Aspartate aminotransferase	45 unit/L	15 - 37 unit/L
Alanine aminotransferase	19 unit/L	8 - 62 unit/L
Alkaline phosphatase	70 unit/L	45 - 117 unit/L
Coombs test	Positive	
IgG antibodies	Positive	
C3 complement	85 mg/dL	90 - 180 mg/dL
C4 complement	11 mg/dL	10 - 40 mg/dL

**Table 2:** Infectious and autoimmune workup. PCR, polymerase chain reaction, HIV, human immunodeficiency virus, Ab, antibody, Ig, immunoglobulin, Ag, antigen, CMV, cytomegalovirus, EBV, Epstein-Barr virus, HSV, herpes simplex virus, ADAMTS13, a disintegrin-like and metalloproteinase with thrombospondin motif, ANA, antinuclear antibody, C-ANCA, antineutrophilic cytoplasmic antibody, P-ANCA, perinuclear anti-neutrophil cytoplasmic antibodies, SS-A/B, Sjorgren syndrome A and B antibodies, Anti-dsDNA, anti-double-stranded DNA.

Biochemical parameter	Patient values
Babesiosis PCR	Negative
HIV	Negative
Hepatitis A total Ab	Positive
Hepatitis A IgM	Negative
Hepatitis B core Ab	Positive
Hepatitis B surface Ab	Positive
Hepatitis B surface Ag	Negative
Hepatitis C	Negative
Mycoplasma IgM	Low positive
Mycoplasma IgG	Positive
Leptospirosis Ab	Negative
CMV	Negative
EBV	Negative
Varicella IgG	Negative
Mumps IgM/IgG	Negative
HSV IgM	Negative
HSV IgG	Positive
ADAMTS13	Negative
ANA	Negative
C-ANCA	Negative
P-ANCA	Negative
SS-A/B	Negative
Anti-dsDNA	Negative
Anti-Smith Ab	Negative
Anti-cardiolipin Ab	Negative

Initial treatment included IVIG 1 g/kg q24h for two days and dexamethasone 40 mg IV daily for four days, with empiric doxycycline with a transient increase in platelets to 48 x 103 /µL. Rituximab with entecavir prophylaxis was added on day 4 due to insufficient response to IVIG and because hepatitis B core antibody was detected. Additionally, the patient was transitioned from dexamethasone to prednisone (1 mg/kg). Course was complicated with altered mentation, hypothermia, hypoxia, hyperbilirubinemia, impaired hepatic and renal functions, and worsening thrombocytopenia and anemia requiring endotracheal intubation with mechanical ventilation, forced-air warming, and transfusion with two units of warmed blood and one unit of warmed platelets. Despite all interventions, critical anemia, thrombocytopenia, and obtundation persisted, and one session of plasmapheresis was undertaken with dramatic improvement in lactate level and mental status allowing extubation despite minimal improvement in anemia and platelets. Sutimlimab was initiated on day 6, and rituximab continued. Rituximab was continued weekly for four doses. Romiplostim was added on day 9 for persistent thrombocytopenia. Platelets slowly rose, allowing discharge with Hgb 7.9 g/dL and platelet 624 x 103 /µL following a total of four doses of rituximab, two doses of romiplostim, and two doses of sutimlimab. Hemolysis labs on discharge included a haptoglobin <6 mg/dL, lactate dehydrogenase 294 unit/dL, total bilirubin 2.2 mg/dL, and direct bilirubin 0.9 mg/dL.

Initial treatment included IVIG 1 g/kg q24h for two days and dexamethasone 40 mg IV daily for four days, with empiric doxycycline with a transient increase in platelets to 48 x 103 /µL. Rituximab with entecavir prophylaxis was added on day 4 due to insufficient response to IVIG and because hepatitis B core antibody was detected. Additionally, the patient was transitioned from dexamethasone to prednisone (1 mg/kg). Course was complicated with altered mentation, hypothermia, hypoxia, hyperbilirubinemia, impaired hepatic and renal functions, and worsening thrombocytopenia and anemia requiring endotracheal intubation with mechanical ventilation, forced-air warming, and transfusion with two units of warmed blood and one unit of warmed platelets. Despite all interventions, critical anemia and thrombocytopenia and obtundation persisted, and one session of plasmapheresis was undertaken with dramatic improvement in the lactate level and mental status allowing extubation despite minimal improvement in anemia and platelets. Sutimlimab was initiated on day 6, and rituximab continued. Rituximab was continued weekly for four doses. Romiplostim was added on day 9 for persistent thrombocytopenia. Platelets slowly rose, allowing discharge with Hgb 7.9 g/dL and platelet 624 x 103 /µL following a total of four doses of rituximab, two doses of romiplostim, and two doses of sutimlimab. Hemolysis labs on discharge included a haptoglobin <6 mg/dL, lactate dehydrogenase 294 unit/dL, total bilirubin 2.2 mg/dL, and direct bilirubin 0.9 mg/dL. 

## Discussion

AIHA is a condition caused by autoantibodies against the erythrocytes, resulting in red blood cell destruction. AIHA is classified as warm or cold antibody types depending on the thermal reactivity of the autoantibodies, and rarely, a mixture of both is identified. Diagnosis is routinely established upon direct antiglobulin testing and evaluation of peripheral smear, in which mixed-type autoimmune hemolytic anemia demonstrates the presence of IgG, complement C3, and cold agglutination. ITP is characterized by low platelet counts and purpura and is a diagnosis of exclusion after ruling out other causes of thrombocytopenia. There have been few reported cases of mixed-type AIHA following SARS-CoV-2 infection, and fewer still of both mixed-type AIHA and ITP.

Plasmapheresis involves removing the patient’s plasma including plasma components such as antibodies. Plasma is typically exchanged for an albumin solution and/or fresh frozen plasma. In this case, plasma was replaced with albumin. IVIG is a pooled antibody made from multiple donors. It is used to block the Fc receptor. Rituximab is a CD-20 directed monoclonal antibody that decreases antibody production. Sutimlimab was approved on 02/2022 for cold AIHA. It blocks the C1s enzyme and prevents the propagation of the classical complement pathway.

The patient presented with mixed AIHA and critical thrombocytopenia. Platelets remained low despite corticosteroids, rituximab, plasma exchange, and sutimlimab. The underlying cause remains obscure. Differentials include COVID-19, rhinovirus, Paxlovid, or occult processes. There are few published reports of COVID-19-associated immune RBC destruction. Jacobs et al. conducted a systematic review of 50 patients who developed hemolytic anemia after COVID infection [7]. Eighteen patients had cold autoimmune hemolytic anemia, 14 patients had warm autoimmune hemolytic anemia, four patients had Evans Syndrome, and the rest were unclassified. Additionally, SN comprehensive clinical medicine reported a systematic review of thrombocytopenia secondary to COVID-19 [1]. In this study, 45 cases of new-onset ITP in COVID-19 patients were described. Seventy-one percent of these patients were found to be elderly (over 50 years old), and 75% of the cases had moderate to severe COVID-19. A few other publications mentioned COVID-19-related Evans Syndrome, which mostly affected younger individuals [8]. Most of these patients survived with just dexamethasone, IVIG, and rituximab. However, the patient was refractory to these treatments but responded to subsequent treatment with sutimlimab, romiplostim, and continued rituximab.

## Conclusions

This is a case report of a 30-year-old male who developed mixed-type AIHA and ITP following SARS-CoV-2 infection and was later diagnosed with Rhinovirus/Enterovirus and mycoplasma. The patient was treated with Paxlovid for COVID-19 and subsequently received standard ITP and AIHA therapies, including intravenous immunoglobulin, glucocorticoids, rituximab, and sutimlimab. Paxlovid is currently approved for patients at high risk for progression to severe COVID-19. In this case, Paxlovid was given in the absence of a recognized high risk of hospitalization or death. This case highlights the hematological complications that can occur in COVID-19 patients and the importance of prompt diagnosis and treatment.
